# Risk perception after genetic counseling in patients with increased risk of cancer

**DOI:** 10.1186/1897-4287-7-15

**Published:** 2009-08-23

**Authors:** Johanna Rantala, Ulla Platten, Gunilla Lindgren, Bo Nilsson, Brita Arver, Annika Lindblom, Yvonne Brandberg

**Affiliations:** 1 Department of Clinical Genetics, Karolinska University Hospital, L5:03, S-17176 Stockholm, Sweden; 2 Department of Oncology and Pathology, Karolinska University Hospital, S-17176 Stockholm, Sweden; 3 Department of Oncology and Pathology, Karolinska Institute, Z4:01 S-17176 Stockholm, Sweden; 4 Department of Oncology and Pathology, Karolinska Institute, Karolinska University Hospital, S-17176 Stockholm, Sweden

## Abstract

**Background:**

Counselees are more aware of genetics and seek information, reassurance, screening and genetic testing. Risk counseling is a key component of genetic counseling process helping patients to achieve a realistic view for their own personal risk and therefore adapt to the medical, psychological and familial implications of disease and to encourage the patient to make informed choices [1,2].

The aim of this study was to conceptualize risk perception and anxiety about cancer in individuals attending to genetic counseling.

**Methods:**

The questionnaire study measured risk perception and anxiety about cancer at three time points: before and one week after initial genetic counseling and one year after completed genetic investigations. Eligibility criteria were designed to include only index patients without a previous genetic consultation in the family. A total of 215 individuals were included. Data was collected during three years period.

**Results:**

Before genetic counseling all of the unaffected participants subjectively estimated their risk as higher than their objective risk. Participants with a similar risk as the population overestimated their risk most. All risk groups estimated the risk for children's/siblings to be lower than their own. The benefits of preventive surveillance program were well understood among unaffected participants.

The difference in subjective risk perception before and directly after genetic counseling was statistically significantly lower in all risk groups. Difference in risk perception for children as well as for population was also statistically significant. Experienced anxiety about developing cancer in the unaffected subjects was lower after genetic counseling compared to baseline in all groups. Anxiety about cancer had clear correlation to perceived risk of cancer before and one year after genetic investigations.

The affected participants overestimated their children's risk as well as risk for anyone in population. Difference in risk perception for children/siblings as for the general population was significant between the first and second measurement time points. Anxiety about developing cancer again among affected participants continued to be high throughout this investigation.

**Conclusion:**

The participant's accuracy in risk perception was poor, especially in low risk individuals before genetic counseling. There was a general trend towards more accurate estimation in all risk groups after genetic counseling. The importance of preventive programs was well understood. Cancer anxiety was prevalent and associated with risk perception, but decreased after genetic counseling.

[1] National Society of Genetic Counselors (2005), Genetic Counseling as a Profession. Available at  (accessed November 25th 2007)

[2] Julian-Reynier C., Welkenhuysen M-, Hagoel L., Decruyenaere M., Hopwood P. (2003) Risk communication strategies: state of the art and effectiveness in the context of cancer genetic services. Eur J of Human Genetics 11, 725-736.

## Background

There is increasing public demand for oncogenetic counseling. Today counselees are more aware of genetics and seek more information, reassurance, screening and genetic testing. For the oncogenetic clinics, it is challenging to provide individualized counseling in the way that it contains all relevant information and fulfill the specific needs and requests of every patient. Risk information is a key component of this process. It is intended to help patients gain realistic views of their personal risk and adapt that risk to the medical, psychological and familial implications of cancer. Information about cancer risk facilitates confidence in making choices about the need of surveillance interventions and in choosing between different control programs and prophylactic procedures. Risk information can be experienced as severe or harmful, and therefore needs to be adapted to the patient's ability and willingness to cope [1,2].

Risk counseling integrates assessments of the: (1) probability that a genetic susceptibility to cancer exists within the family, (2) probability that the patient has inherited predisposing gene(s) and (3) risk that the patient develops a genetically inherited cancer. These questions concern not only the patient but also close family members and offspring [2,3]. Risk information is often based on family history and the probability of inheritance of cancer ranging from no hereditary cancers to the inevitability of intervention for survival, although such extremes are rare [4]. Adequate risk information also includes information about the condition in question, modifying risk factors, medical managements and treatment options. The effectiveness of preventive procedures must be addressed along with information about benefits and limitations related to testing, practical information of a positive or negative test result and risk management. Information about available support groups must also be provided [5].

Hereditary cancer syndromes have become better understood, but surprisingly many patients overestimate their risk for cancer and experience anxiety of developing the disease. Questions concerning risk perceptions in individuals and families and questions about the effect of genetic counseling on risk comprehension are therefore of particular relevance [6].

The accuracy of risk estimations varies between studies [7]. Some reports state that only approximately 25% of patients have accurate risk perception while 50% of patients tend to overestimate their risk for cancer [8]. Another study indicated that 50% of the non-affected patients were accurate in risk perception at baseline before genetic counseling and the remaining 50% was divided into equal proportions of underestimations and overestimations [9]. As many as 67% of participants in another study reported an accurate risk, while among those with inaccurate risk perceptions equal number of respondents over or underestimated their risk for cancer [10]. In a Norwegian study 41% of the participants believed that they had the same or lower risk of developing cancer than their peers of same age and gender [11].

Oncogenetic counseling was established at the Karolinska University Hospital in 1990 in order to offer cancer risk estimates and preventive programs to patients. We have found no previous study that prospectively examines in detail various aspects of risk perception and also associate risk perception to cancer anxiety. The present study was conducted (1) to evaluate oncogenetic counseling and (2) to learn from patient experiences in order (3) to improve the quality of genetic counseling. Risk estimates and anxiety about cancer in unaffected and affected individuals from this study are the focus of this article.

The following topics were addressed: (1) How do **unaffected **patients (individuals with no previous cancer diagnosis) estimate their own, their sibling's/children's and the general population's risk of cancer before genetic counseling, immediately after, and one year after completed genetic investigations as compared to the risk estimated by an oncogenetic physician?, (2) How do unaffected patients perceive their own risk compared to the risk for the general population?, (3) Is there a difference in risk perception among unaffected individuals if they would hypothetically participate in a preventive programs compared to no preventive programs?, (4) Are there differences in cancer anxiety about developing cancer before and after genetic counseling?, (5) Is there a correlation between perceived risk and anxiety about cancer?, (6) How do **affected **patients (individuals with a cancer diagnosis) estimate their siblings/children's risk and the general population's risk for cancer before, after genetic counseling and one year after completed genetic investigations?, (7) Are there differences in cancer anxiety about developing cancer again before and after genetic counseling?

## Materials and methods

### The oncogenetic procedure at the Karolinska University Hospital

Patients were referred to oncogenetic counseling at the Karolinska University Hospital based on the basis of family history of breast-, ovarian-, colorectal-, endometrial or gastric cancer either on their own initiative or by their general practitioners. During the first consultation an oncogenetic nurse informed the patients about the counseling procedures and established a family pedigree in collaboration with the patient. Prior to the meeting with a clinical geneticist, cancer diagnoses in the family were confirmed through medical records and/or death certificates. The genetic counseling process included a review of: (1) the family history of cancer, (2) the cancer risk estimation, (3) the possibility of genetic testing and, (4) the available surveillance programs. At the clinic counselors followed routines in an effort to guarantee that every patient received counseling with the same quality. All patients received a written summary after counseling.

### Participants

Only patients with no previous genetic consultation in the family were eligible to participate in the study. Persons who had previously been informed about or done pre-symptomatic testing were not included in this study. A total of 310 patients who were referred to the Department of Oncogenetic at the Karolinska University Hospital during the course of one year and they were invited to participate in a questionnaire study. Questionnaire data was collected during a study period of three years. All participants had good Swedish comprehension. Of the 310 invited patients, 254 (82%) showed interest in genetic investigations while no contact was obtained from 56 (18%) of the patients after referral. None of the participants were given results of genetic testing during the study period.

The individual risk of developing cancer (the objective risk) was estimated for every patient by a geneticist (AL). The mean risk for cancer in each risk group can be roughly estimated as the mean value of both breast cancer and colon cancer risks, because these cancer types are the most prevalent ones. Thus, the unaffected patients were categorized into four risk groups: same as the general population 5-11% risk, low 12-23%, moderate 24-45% and high risk >45%. The number of unaffected patients was too small to be analyzed in subsets according to cancer diagnoses. Affected patients were analyzed as one group because they were considered to have risk for recurrence, for developing metastatic disease and risk for developing another primary cancer.

### Questionnaires

The questionnaires were jointly designed by a geneticist (AL), a psychologist (YB), a nurse (UP) and a bachelor of public health (GL). The questionnaires were tested on four patients and found feasible for the study. No formal reliability testing or validation was performed.

The patients were given separate questionnaires depending on whether or not they were previously diagnosed with cancer, i.e., "affected" or "unaffected". The type of cancer in question was the cancer type the individual had a family history of. The risk in this context means lifetime risk.

Questionnaires 1 (before consultation) and 2 (after consultation) included five questions about risk perception and anxiety that unaffected patients experience about developing cancer. The first question concerned how patients estimate their risk of cancer compared with the risk for anyone in the general population. The patients were asked to answer the questions of risks according the type of cancer diagnosis in the family. The respondents were included only if the patients gave estimations for the type of cancer which existed in the family. Responses were given on a five point scale from "Much lower" to "Much higher". The following items elicited patients' estimation of their own, siblings/children's and general population's risk of cancer on a visual analogue scale with the end points "No risk" to "100% risk". The last question concerned anxiety about developing cancer scored on a scale "Not at all" to "Very much". In questionnaire 3 (one year after genetic investigations) unaffected patients estimated their risk of developing cancer compared to the risk for anyone in the general population. They estimated their own, their siblings/children's and the general populations hypothetical risk of developing cancer depending on whether they hypothetically would or would not participate in preventive programs. Question concerning anxiety about developing cancer was the same as in questionnaire 1 and 2.

In the questionnaire designed for affected patients, the question about their own perceived risk of developing cancer did not exist, but otherwise risk perception questions were the same as for unaffected individuals in questionnaire 1 and 2. A question about the anxiety about developing cancer was formulated as anxiety about developing cancer again. In questionnaire 3, affected patients estimated the hypothetical risk to their sibling's/children's and the general population based on whether or not they hypothetically would participate in preventive programs.

### Evaluation of objective risk

The empirical cancer risk in the family, based on family history, and personal risk for each patient were assessed by a geneticist (AL). Risk was defined in four categories; "The same risk as population", "Low risk", "and Moderate risk" and "High risk". This assessment was made after the patients met with one of the counselors at the department of clinical genetics. For breast cancer empirical risk estimations Claus model were used as guideline. For colon cancer empirical risk estimations were based on international publications [12-19].

### Procedure

The questionnaires were completed at three points of assessment: immediately before genetic counseling, one week after initial genetic counseling and one year after completion of genetic investigations. The participants were sent a questionnaire with information about the study immediately before the first contact and they returned the questionnaire at the first counseling appointment. The second questionnaire was sent together with a prepaid return envelope to participants one week after the visit. The third and final questionnaire was sent with a prepaid return envelope to the participants one year after completion of genetic counseling. The participants who did not return the questionnaire received three remainders encouraging them to continue in the study.

### Ethical Aspects

The study was performed in accordance with Swedish law (2003:460) and approved by the local Ethics Committee, D:nr 2005/566-31/1.

### Statistical analysis

Data analyses were performed with the Statistical Package for the Social Sciences (SPSS) or Statistica 8 program. Descriptive statistics were generated to arrange the participants by age, gender and presence of cancer and risk perception.

Student's t-test (paired and unpaired) and ANOVA factorial designs were used to analyze the differences between measuring time points and group differences. Spearman's rho test was used to test the correlation between the risk perceptions.

## Results

### Characteristics of participants

**Figure 1:**
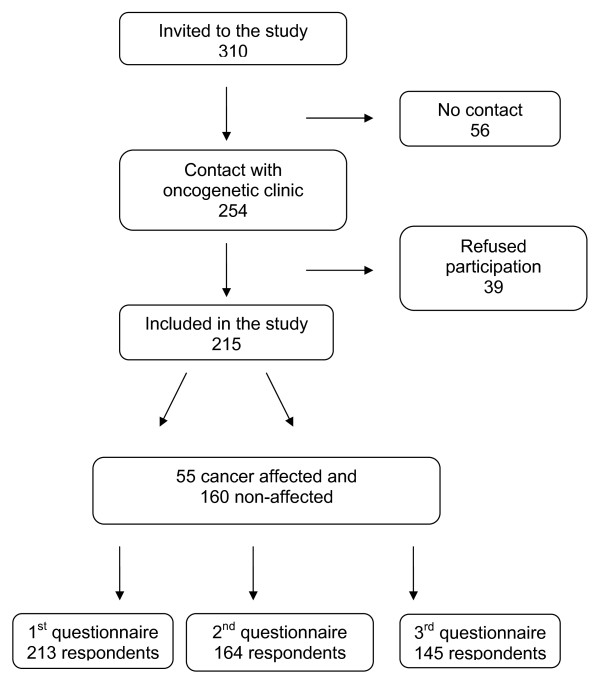
**Study material**. Flow chart illustrates the included and excluded patients and the number of respondents in each questionnaire.

A total of 215 patients (85% of eligible patients) returned at least one questionnaire and were included in the study (Figure 1). A total of 213 (99% of included patients) returned the first questionnaire and 164 (76% of included patients) returned the second questionnaire. The last questionnaire generated 145 respondents (67%).

Gender and age were used as socio-demographic variables in the present analyses. There were no statistically significant differences in gender, age or medical characteristics between respondents and non-respondents. The mean age among non-respondents was 46 (SD = 10.2) and among respondents 45 (SD = 10.5) at initial contact. Eighteen percent (*n *= 7) of non-respondents and 11% (*n *= 23) of respondents were male. Eighteen percent (n = 7) of non-respondents were affected; the corresponding figure for respondents was 26% (n = 55) (p = 0.321).

Affected participants were statistically significantly older (mean age 54, *n *= 55) than unaffected (mean age 42, *n *= 160) [df (1, 213), t = -6.384, p < 0.001]. A total of 47 (25%) female participants were affected, the corresponding figure for men was 8 (35%).

A total of 93 (43%) participants were referred to oncogenetic counseling by their own initiative, 110 (51%) by general practitioners, 14 (7%) by a relative and 9 (4%) by "somebody else". The participants could choose more than one response alternative to this item and 12 patients answered that they participated in counseling both on their own initiative and on the request of a relative.

A total of 156 patients (60%) had no previous history of cancer and were categorized as "unaffected" The unaffected participants were referred to genetic counseling due to a family history of breast- (*n *= 104), ovarian- (*n *= 5), colorectal- (*n *= 43) endometrial (*n *= 1) or gastric- (*n *= 3) cancer. Information about cancer type and objective risk was missing in four unaffected participants because they returned the first questionnaire partly completed and never attended genetic counseling. However, responses from these four participants were used in some of the analysis.

The affected participants were referred to oncogenetic counseling based on a family history of breast- (*n *= 22) ovarian- (*n *= 9), colon- (*n *= 15) and endometrial cancer (*n *= 1). For 8 patients the information about cancer type is missing because these participants only returned the first questionnaire partly filled and never attended genetic counseling. These 8 responses were used in some of the analyses.

### Risk Perception of non affected participants

#### Own Risk Perception

Whether the patients were referred to oncogenetic counselling on their own initiative (*n *= 61) or on general practitioners (*n *= 62) did not appear to influence their own risk perception (mean risk estimation 61.9% versus 59.7%, p = 0.567) nor risk perception for children/siblings (50.6% versus 45.8%, p = 0.299) or risk estimates for general population (29.6% versus 28.9%, p = 0.841).

The type of cancer history in the family did not appear to influence the risk perception. The difference in risk perception between the two most prominent cancer types was not statistically significant before genetic counseling; mean risk perception in breast cancer families was 62.5% (*n *= 93) and in colon cancer families 56.1% (*n *= 34), (p = 0.132). After genetic counseling the mean risk perception in colon cancer families was 45.1% (*n *= 29) and 48.0% in breast cancer families (*n *= 69), (p = 0.593). There were no statistically significant differences in risk perception for children either between individuals with breast cancer (47.5%, n = 101) or colon cancer (51.4%, n = 43), (p = 0.379) in the family.

As the number of individuals in each cancer type group was small, the individual responses independent on cancer diagnosis were combined in order to increase statistical power.

**Figure 2:**
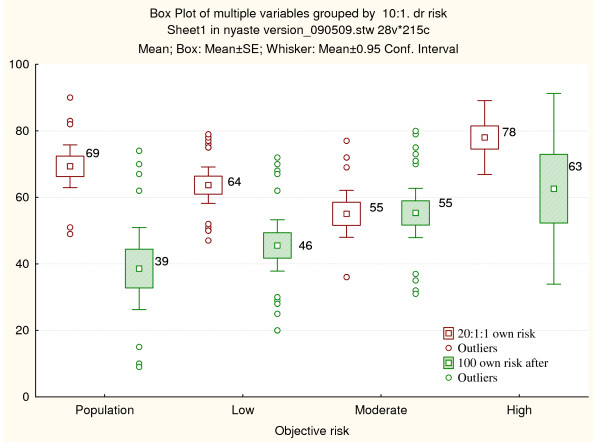
**Own subjective risk perception**. Own subjective risk perception in unaffected patients before and after genetic counseling illustrated separately stratified according to objective risk estimated by the clinical geneticist (population, low, moderate and high risk). The mean, standard, and 95% CI and outliers are shown.

Before genetic counseling all of the risk groups subjectively estimated the risk as higher than the objective risk. Subjective estimations in all participant groups ranged between 55% and 78% (Figure 2). The participants with a similar risk as the population overestimated their risk the most. Individuals with moderate and high risk for cancer were more accurate in subjective risk estimations.

**Figure 3:**
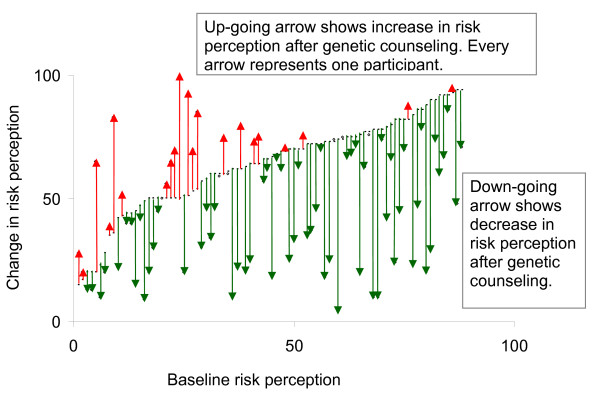
**Change in risk perception among unaffected participants**. Increases and decreases in perceived risk after genetic counseling for all unaffected patients who responded to the first and second questionnaires (*n *= 90). Up-going arrow shows increase in risk perception after genetic counseling and down-going arrow show decrease in risk perception after genetic counseling. When two dots are presented but no arrow, it means that the risk perception has not changed. Every arrow represents one patient.

A total of 91% (n = 82) of unaffected participants altered their risk perception after genetic counseling (Figure 3). Of them, 68% (n = 61) showed a decrease in risk perception while 23% (n = 21) reported increased risk perception. Nine percent (n = 8) had the same risk perception at both points of assessment.

The difference in subjective risk perception before and directly after genetic counseling was statistically significant [df = (1, 90), t = 4.73, p < 0.001]. Difference in risk perception for children was also statistically significant [df = (1, 78), t = 5.05, p < 0.001], as well as difference in risk perception for the population [df = (1, 82), t = 3.50, p =< 0.001]. The perception of one's own cancer risk was more accurate directly after genetic counseling in the groups with the same risk as the general population and low risk (Figure 2). Moderate risk individuals did not show any change in risk perception after genetic counseling. In the group with the same risk as the general population, risk perception was reduced from 69% to 39%, although still almost four times higher compared to their objective risk. Low risk participants reduced their risk perception from 64% to 46%, still more than two times higher than their objective risk. Individuals in the moderate risk group overestimated their risk by 20% compared to their objective risk.

One year after genetic investigations, the unaffected individuals were asked about their perceived risk contingent upon participation in prophylactic interventions such as check-ups (i.e. mammography and colonoscopy) or preventive surgery. The hypothetical risk perception was lower if the participants would take part into preventive programs than without such interventions (37% versus 56%) [df = (1, 80), t = 7.57, p < 0.001]. The moderate and high-risk individuals reported the largest difference in risk estimation depending on whether or not they considered attending preventive programs (moderate risk individuals without control 62% versus with check-ups 39% and high risk individuals without check-ups 74% versus with control 31%.

There were no statistically significant differences in mean risk estimation between individuals with breast or colon cancer risks if they hypothetically would participate in preventive programs.

### Risk perception for sibling/children and general population

Risk perception for children was independent of the type of cancer in the family. Individuals in breast cancer families had a mean risk estimation for children/siblings of 48.9% before genetic counseling and colon cancer families had 48.9% (p = 0.761). After genetic counseling the risk estimation were 34.9% versus 41.0% (p = 0.534). Subsequently, results from the total sample have been analyzed together.

**Figure 4:**
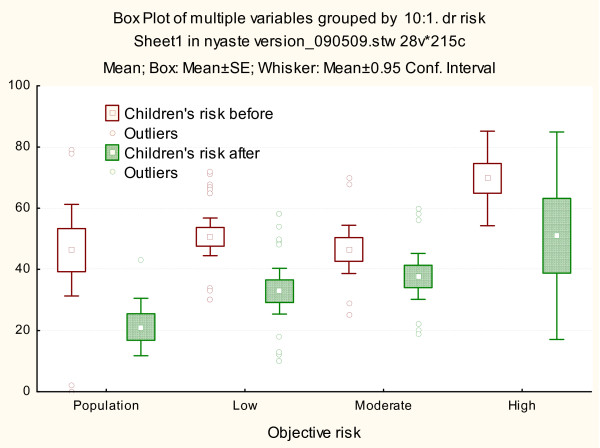
**Risk perception form siblings/children**. Risk perception for sibling and children before and after genetic counseling in unaffected patients illustrated separately stratified according to objective risk estimated by the clinical geneticist (population, low, moderate and high risk). The mean, standard error, 95% CI and outliers are shown.

**Figure 5:**
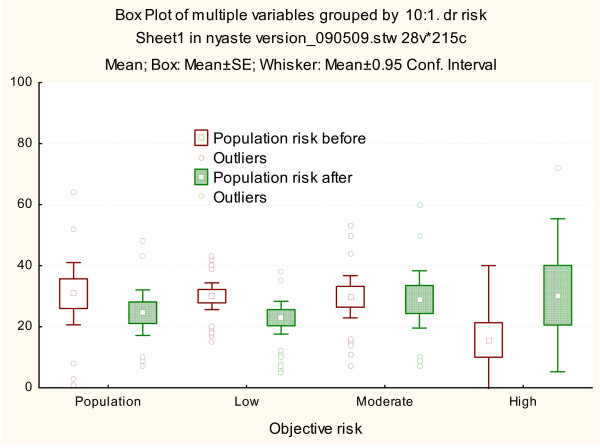
**Risk perception for the general population**. Risk perception for the general population before and after genetic counseling in unaffected patients illustrated separately stratified according to objective risk estimated by the clinical geneticist (population, low, moderate and high risk). The mean, standard error, 95% CI and outliers are shown.

Perceived risk for siblings/children was significantly lower than personal risk estimation [df = (1, 119, t = 5.89, p < 0.001], but was also overestimated with a mean estimation of 48.1% before and 33.9% after genetic counseling, statistical significant over time (p < 0.001). In the groups with the same risk as the general population and low risk, the participants showed biggest differences in risk perception before and after genetic counseling (Figure 4). Risk perception for the general population was also overestimated, but lower than the risk perceived for children [df = (1, 146) t = 8.00, p < 0.001] (Figure 5).

One year after genetic investigations the unaffected individuals were asked about their children's/siblings and general population's hypothetical risk if they **would not **participate in preventive programs. Risk perception was 46.9% for children and for the general population 29.0%. The cancer type or the objective risk did not have influence in risk perception.

### Perceived risk of developing cancer compared to the risk for anyone in the general population

After genetic counseling, more participants (55%) in the population risk group reported that they had an equal risk of developing cancer compared to anyone in the general population compared to before genetic counseling (4.3%) (Table 1) A total of 96% of participants in the population risk group perceived the risk as higher than or as much higher as the risk for anyone in the general population. After genetic counseling however, this proportion decreased to 45%. This difference was statistically significant [df = (1, 20), t = -3.23, p = 0.005]. None of the other risk groups showed such a clear change in risk perception.

**Table 1:** Risk perception compared to anyone in general population

	Before Counseling %	After Counseling %	1 year after Surveillance program %	1 year after **No **surveillance program %
Population	*n *= 23	*n *= 22	*n *= 11	*n *= 11
Much higher (=5)	17.4%	13.6%	-	-
Higher (=4)	78.3%	31.8%	18.2%	63.6%
Equal (=3)	4.3%	54.6%	36.4%	27.3%
Less (=2)	-	-	45.4%	-
Much less (=1)	-	-	-	9.1%

Mean value at scale 1-5 (SD)	4.13 (0.33)	4.08 (0.66)	2.8 (0.64)	3.4 (0.72)

Low	*n *= 51	*n *= 47	*n *= 37	*n *= 39
Much higher (=5)	21.6%	17%	5.5%	12.8%
Higher (=4)	64.7%	70.2%	40.5%	76.9%
Equal (=3)	13.7%	12.8%	27%	7.7%
Less (=2)	-	-	27%	2.6%
Much less (=1)	-	-	-	-

Mean value at scale 1-5 (SD)	4.1 (0.41)	4.05 (0.35)	3.22 (0.85)	4.00 (0.27)

Moderate	*n *= 41	*n *= 38	*n *= 35	*n *= 35
Much higher (=5)	26.8%	34.3%	2.9%	31.4%
Higher (=4)	63.4%	60.5%	45.7%	62.9%
Equal (=3)	7.3%	2.6%	20%	5.7%
Less (=2)	2.5%	-	25.7%	-
Much less (=1)	-	2.6%	5.7%	-

Mean value at scale 1-5 (SD)	4.15 (0.49)	4.23 (0.56)	3.17 (0.9)	4.27 (0.44)

High	*n *= 5	*n *= 5	*n *= 5	*n *= 5
Much higher (=5)	60%	11.1%	-	60%
Higher (=4)	40%	66.7%	40%	20%
Equal (=3)	-	22.2%	20%	20%
Less (=2)	-	-	40%%	-
Much less (=1)	-	-	-	-

Mean value at scale 1-5 (SD)	4.6 (0.48)	4.17 (0.28)	3.00 (0.8)	4.4 (0.72)

Risk for developing cancer compared to risk for anyone in general population in different risk groups on a scale from "much higher" to "much less" assessed by unaffected participants. The values are the proportion of patients in each risk category, according to perceived risk compared to the risk for anyone in the general population.

The correlation between risk perception for anyone in the general population and for personal risk was statistically significant [r = 0.450, p < 0.001] when measured on the first occasion and also after genetic counseling [r = 0.462, p < 0.001]. One year after genetic investigations the correlation between risk perception for themselves and for the general population was also statistically significant when asking hypothetically the patients if they would participate in a preventive program [r = 0.380, p < 0.001] or if they would not participate [r = 0.460, p < 0.001].

### Experienced anxiety about developing cancer

**Figure 6:**
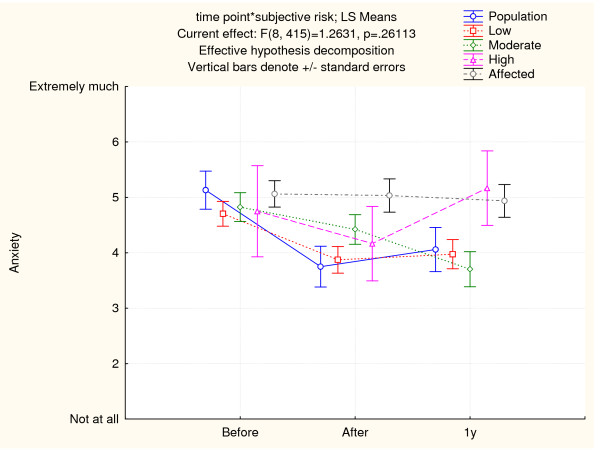
**Cancer anxiety**. Anxiety in unaffected an affected patients before, after genetic counseling and 1 year after genetic investigations is illustrated. Unaffected patients are stratified according to objective risk estimated by the clinical geneticist (population, low, moderate and high risk). The mean and standard errors are shown.

Experienced anxiety about developing cancer according to the risk group and point of time for assessment is displayed in Figure 6. The degree of experienced anxiety about developing cancer in the unaffected subjects was lower after genetic counseling compared to the baseline in all other groups [df = (1, 105), t = 5.79, p < 0.0001], except in high risk individuals. The difference in anxiety immediately after genetic counseling and one year after was not statistically significant.

### Correlation between experienced anxiety about developing cancer and estimated risk for cancer

The correlation between perceived risk and anxiety about developing cancer was statistically significant before genetic counseling [*n *= 140, r = 0.412, p < 0.001], after genetic counseling [*n *= 101, r = 0.416, p < 0.001] and one year after genetic investigations among unaffected patients. One year after genetic investigations, the question was presented depending on what the patients thought the risk would be if they would participate in preventive programs [*n *= 81, r = 0.346, p = 0.002] and if they would not participate in preventive programs [*n *= 81, r = 0.414, p < 0.001].

### Risk perception of affected participants

Whether or not the subjects were referred to oncogenetic counseling on their own initiative or on general practitioners initiative did not influence risk perception for children (mean risk estimation 47.7% versus 58.7%, p = 0.180 nor the risk perception for the general population (mean risk estimation 30.4% versus 31.9%, p = 0.820). The affected subjects did not estimate their own risk due to previous cancer history.

Risk perception for children before genetic counseling between the two most prominent cancer types were not statistically significant, 46.9% in breast cancer families and 68.3% in colon cancer families (p = 0.066). Thus, the results from all affected individuals independent the cancer type are analyzed together.

### Risk perception for siblings and children and for the general population

The affected participants overestimated their children's risk as well as the risk for anyone in the general population. The difference in risk perception for children/siblings was statistically significant before (mean 57.1%) versus immediately after genetic counseling (mean 48.2%) [df = (1, 22) t = 2.04 p = 0.054]. Risk perception remained about the same one year after genetic investigations [df = (1, 16), t = -1.04, p = 0.313].

Differences in risk perception for the general population were also statistically significant before (mean 37.2%) versus after genetic counseling (mean 29.5%) [df = (1, 22), t = 2.54, p = 0.019]. There was no difference in risk perception between the second and third assessment for the general population.

### Anxiety about developing cancer again

Anxiety about developing cancer again among affected participants continued to be high throughout this investigation (Figure 6). Anxiety was estimated on a scale from very much (7) to not at all (1). The mean anxiety was 5.06 (SD = 1.72) before genetic counseling, 5.03 (SD = 1.69) after and 5.03 (SD = 1.54) one year after genetic investigations. There was no statistically significant difference between any of the assessment points.

## Discussion

The current study reveals the personal risk perception related to the objective cancer risk and associates the personal risk perception to the risk for children and the general population. The results indicate that before genetic counseling subjective risk was overestimated by the majority of participants, especially among those with low objective risk. Risk perception, however, were more accurate after genetic counseling among the low risk subjects that had been most worried. The perceived risk for siblings, children and the general population was overestimated by both affected and unaffected patients, but was also more accurately assessed after genetic counseling. Participating in preventive programs was considered to result in decreased risk. Cancer anxiety in unaffected patients decreased after genetic counseling, but was still associated with perceived risk throughout the year following genetic investigation. Cancer anxiety in affected patients did not appear to be influenced by genetic counseling.

An accurate perception of one's own risk and level of anxiety are important. In the literature, data indicate that increasing perception or personal vulnerability, such as greater anxiety, is associated with a higher use of health care actions [20]. Patients who underestimate their risk for cancer may not give sufficient attention to preventive procedures and overestimation of risk might lead to anxiety and unnecessary use of medical services [21]. Participants with a diagnosis of cancer reported higher levels of anxiety, demonstrating quantitatively that cancer diagnosis affects general well-being. This must be taken into consideration when counseling affected individuals [22].

Participants perceived their own risk as being higher than the risk for anyone in the general population, results which are in concordance with a previous Swedish study [22]. Even those with an objective risk similar to the general population overestimated their risk. This finding is not surprising considering that they attended the clinic due to a subjective perception of increased risk for cancer or that their general practitioners revealed concern about the risk. The risk perceptions were, however, more accurate in these groups after genetic counseling, although still higher than the actual estimation determined by the genetic counselor.

In 1993 a pilot study of risk perception indicated that only 11% of women in a family affected with breast cancer were able to identify the accurate risk for women in the general population. Less than half were able to identify their own lifetime risk within 50% range of the counselor's estimation [23]. A follow-up study reported that 33% of participants had the correct risk estimation for the general population after genetic counseling and 41% estimated their own risk accurately [24]. In a Swedish study of women who were considering a prophylactic mastectomy due to a hereditary increased risk for breast cancer but with no previous cancer history, 25% overestimated their risk for cancer by more than 20% although 28% of women underestimated their risk by more than 20% [22].

In the present study, a radical overestimation of the risk was shown in unaffected individuals with low or similar risk as the general population. This finding is in concordance with a German study showing that moderate and low risk individuals made the most marked overestimation [25]. This phenomenon was also reported in a Swedish study of risk perception in individuals at risk for colon cancer who participated in a surveillance program. Among those having slightly increased risk (1-20% lifetime risk), 60% overestimated their risk [26]. Individuals with real increased risk were more accurate in the current study, a similar phenomenon was shown in the German study as well [25]. The previous Swedish study also unexpectedly reported that after genetic counseling the majority of the mutation carriers with an 80% objective lifetime risk underestimated their risk. 61% of subjects estimated their risk as being 40% or less and 36% estimated a 1-20% risk. Only half of the patients having a 40% lifetime risk estimated that risk correctly [26]. Liljegren *et al*. reported that the risk estimations varied widely, but suggested that the most important task in genetic counseling is to ensure that those with increased risk attend surveillance programs and do not suffer from anxiety or depression.

The finding in this study of more accurate risk estimation after genetic counseling is supported by a number of studies [7,27]. However the long-term effect is unclear. In one Swedish study the proportion of accurate risk estimators increased after consultation but decreased again one year after genetic investigations. The proportion of under and overestimators was halved after genetic counseling, but both increased one year after consultation to the level before genetic counseling [28]. In the present study the risk perception did not appear to change from the assessment point after genetic counseling to the one-year assessment. Recall based on cognitive functioning is probably one reason for more accurate risk estimates directly after counseling, whereas long term risk perception is influenced by emotions and thus involves interpretation.

In the present study the unaffected subjects considered their risk to be lower if they considered participation in a preventive program as compared to not participating in such a program. The largest difference in risk perception considering participation in preventive programs was found among moderate and high risk individuals. These participants reported the risk as being 40% lower with a surveillance program. The importance of preventive programs is thus understood and the results demonstrate the importance of giving recommendations and supporting patients to participate in check-up procedures. The most of the participants were not included in preventive programs during the time of the current study. A study about predictive testing for HNPCC post-test delineated that none of the non-carriers and all of the mutation carriers adhered to the recommendations [29]. Many other studies have also shown that knowledge about the benefit of surveillance programs in reducing the risk of cancer is beneficial among patients with increased risk for cancer and that prophylactic measures are appreciated and believed to be effective [30,31].

Experienced anxiety about cancer in the unaffected patients reduced significantly in all risk groups after genetic counseling in accordance with previous studies [11,32]. The anxiety about cancer continued to decrease slightly also one year after genetic investigations except in low risk patients where the level of anxiety remained approximately the same as at the assessment point after genetic counseling. In all risk groups the proportion of individuals with high levels of cancer anxiety tended to decrease immediately after genetic counseling with a further decline one year afterwards. A similar tendency was reported by Bish *et al *[33]; anxiety about developing cancer and perception of the likelihood of being a mutation carrier decreased significantly after genetic counseling. However, the same study reported that general psychological distress did not change over the course of follow-ups before and after genetic counseling. Hopwood *et al *describes that after genetic counseling, women with over- or underestimated risk perception had significantly higher general health distress levels than women with accurate risk-knowledge. As their main concern, the women reported not only risk of breast cancer but even themes of loss, unresolved grief and problems within relationships [34]. Thus a number of factors might affect general distress. General distress was, however, not examined in the present study.

Affected patients had a tendency to overestimate the general population risk for cancer. The same was found in a study of breast cancer concerns in women attending a genetic counseling clinic at a comprehensive cancer center. Affected and unaffected individuals perceived that the women who had already developed cancer are less likely in risk to develop cancer again. When asking the affected patients to recall what they thought their risk was before cancer onset, they estimated the risk of developing breast cancer from 0 to 100% with a mean of 31%. The unaffected women estimated their risk to be approximately 15% with a range 5-100%. The lowest risks were reported by the youngest and by women who have undergone bilateral mastectomy. Many women thought that their risk was confined to the next 10 years, being 33% (mean) for affected with 0-75% range and for unaffected 56% (mean) varying between 0 and 100% [35].

Women with a family history of cancer and overestimation of risk have been shown to report significantly higher breast cancer specific distress [36]. In the present study the affected individuals showed more concerns about cancer and they had higher perceptions of risk for other cancers than non-affected. This is natural because affected individuals have risk for developing recurrence and metastasis disease and also risk for another primary cancer. This indicates the need for more sensitive counseling to affected individuals [34].

The present study has some limitations. The questionnaires were developed by the researchers and not formally validated or reliably tested. Cancer anxiety was assessed by one question which could be considered imprecise. However, it showed changes over time, giving at least an indication that anxiety decreased after genetic counseling. In addition, associations between personal risk estimation and cancer anxiety were revealed. Perceived risk (subjective risk) was assessed by a VAS scale where risk was stated as a point estimate. Objective risk was based on the family pedigree (cancer history) and stated in four categories. This difference might have impacted the findings as small changes in perceived risk were recorded and compared to the wide categories of objective risk.

According to a review on approaches to communicating health risks, numerical information regarding the probability that a health problem occurs is difficult to understand. The patients found risk information through numerical probabilities such as absolute numbers, relative risks, ratio and frequency of events or proportions as too scientific. This problem has been underestimated by clinicians in health care. An alternative way to inform patients about the risks is to give verbal descriptions like "an unlikely event" or "risk higher than average" [37]. This way of conveying the risks could be easier because the patients tend to reformulate the risk information according to personal and family experiences of cancer and relate the risk to lifestyle. They reconstruct the risk according to what they believe about inheritance and what experience of cancer the family has. It might be an attempt to get back control of their destiny or to understand how cancer is inherited via generations or that the patients try to connect the environment with genetics [38]. It has been shown that there is a connection between observed knowledge of hereditary cancer and understanding of personal cancer risk. Thus, the counseling strategies exploit the fact that knowledge derived from experience often takes precedence over an objective risk estimate. Subsequently, communication regarding patient experiences may enhance risk perception [39,8].

## Conclusion

The participant's accuracy in risk perception was poor, especially in low risk individuals before genetic counseling. There was a general trend towards more accurate estimation in all risk groups after genetic counseling. The importance of preventive programs was well understood. Cancer anxiety was prevalent and associated with risk perception, but decreased after genetic counseling.

## Competing interests

The authors declare that they have no competing interests.

## Authors' contributions

The questionnaires were jointly designed by AL, YB, UP and GL. UP collected the questionnaires and participated in study design and coordination. The statistical analyses were performed by JR and BN. JR drafted the manuscript. All authors read and approved the final manuscript.
